# Motion‐corrected simultaneous cardiac positron emission tomography and coronary MR angiography with high acquisition efficiency

**DOI:** 10.1002/mrm.26690

**Published:** 2017-04-20

**Authors:** Camila Munoz, Radhouene Neji, Gastão Cruz, Andrew Mallia, Sami Jeljeli, Andrew J. Reader, Rene M. Botnar, Claudia Prieto

**Affiliations:** ^1^ Division of Imaging Sciences and Biomedical Engineering King's College London London United Kingdom; ^2^ MR Research Collaborations, Siemens Healthcare Frimley United Kingdom; ^3^ PET Centre, St Thomas' Hospital, King's College London & Guys and St Thomas' NHS Foundation Trust London United Kingdom; ^4^ Escuela de Ingenieria, Pontificia Universidad Catolica de Chile Santiago Chile

**Keywords:** cardiac PET‐MR, nonrigid motion correction, coronary MRA, myocardial PET

## Abstract

**Purpose:**

Develop a framework for efficient free‐breathing simultaneous whole‐heart coronary magnetic resonance angiography (CMRA) and cardiac positron emission tomography (PET) on a 3 Tesla PET‐MR system.

**Methods:**

An acquisition that enables nonrigid motion correction of both CMRA and PET has been developed. The proposed method estimates translational motion from low‐resolution 2D MR image navigators acquired at each heartbeat and 3D nonrigid respiratory motion between different respiratory bins from the CMRA data itself. Estimated motion is used for correcting the CMRA as well as the emission and attenuation PET data sets to the same respiratory position. The CMRA approach was studied in 10 healthy subjects and compared for both left and right coronary arteries (LCA, RCA) against a reference scan with diaphragmatic navigator gating and tracking. The PET‐CMRA approach was tested in 5 oncology patients with ^18^F‐FDG myocardial uptake. PET images were compared against uncorrected and gated PET reconstructions.

**Results:**

For the healthy subjects, no statistically significant differences in vessel length and sharpness (*P* > 0.01) were observed between the proposed approach and the reference acquisition with navigator gating and tracking, although data acquisition was significantly shorter. The proposed approach improved CMRA vessel sharpness by 37.9% and 49.1% (LCA, RCA) and vessel length by 48.0% and 36.7% (LCA, RCA) in comparison with no motion correction for all the subjects. Motion‐corrected PET images showed improved sharpness of the myocardium compared to uncorrected reconstructions and reduced noise compared to gated reconstructions.

**Conclusion:**

Feasibility of a new respiratory motion‐compensated simultaneous cardiac PET‐CMRA acquisition has been demonstrated. Magn Reson Med 79:339–350, 2018. © 2017 The Authors Magnetic Resonance in Medicine published by Wiley Periodicals, Inc. on behalf of International Society for Magnetic Resonance in Medicine. This is an open access article under the terms of the Creative Commons Attribution License, which permits use, distribution and reproduction in any medium, provided the original work is properly cited.

## INTRODUCTION

MRI and positron emission tomography (PET) are very promising noninvasive tools for early risk assessment, therapy guidance, and treatment monitoring of coronary artery disease (CAD). Clinical research studies have shown the potential of MRI for the noninvasive assessment of cardiac function, infarct size, and coronary artery stenosis using CINE‐MRI, late gadolinium enhancement, and coronary MR angiography (CMRA), respectively 1. PET has been extensively validated for the accurate assessment of myocardial viability with ^18^F‐FDG (fluorodeoxyglucose) and for the quantification of myocardial perfusion using ^13^N ammonia, ^82^Rb, or ^15^O‐water tracers 2. For instance, it has been shown that the extent and severity of myocardial perfusion defects can be used for risk stratification of CAD patients 3. Additionally, the presence of viable myocardium can be used to guide therapy planning in patients with chronic ischemic disease, given that it has been shown to strongly predict potential functional recovery after revascularization therapy 4. Moreover, the use of ^18^F‐sodium fluoride PET imaging has been demonstrated for identification of ruptured and high‐risk coronary atherosclerotic plaque 5.

The complementary anatomical and functional information provided by recently introduced PET‐MR systems offers great potential for simultaneous assessment of myocardial integrity (viability or perfusion) by PET and coronary lumen integrity by CMRA in a single examination 6. However, image degradation attributed to physiological motion during data acquisition in both modalities remains a major challenge.

Simultaneous PET‐MR systems have made possible the use of MR‐measured motion fields to directly correct the degraded PET data. Several simulation studies have shown that MR‐based PET motion compensation improves lesion detectability and reduces noise levels compared to gated images, while reducing blurring and improving contrast compared with non‐motion‐compensated images for cardiac 7, 8 and abdominal and thoracic 9, 10, 11, 12, 13, 14, 15, 16 applications. Phantom 17, 18, 19, 20 and preliminary patient studies 21, 22, 23, 24, 25 have confirmed these findings.

Current developments for MR‐based PET respiratory motion compensation estimate the motion by either creating a motion model using previously acquired multislice 2D 21, 25 or 3D dynamic MRI (9,11‐14) (so‐called precalibrated motion models) or by applying image‐based registration algorithms to images obtained from a simultaneous retrospectively gated MR acquisition (22–24, see Munoz et al 26 for a comprehensive review). Precalibrated motion model approaches commonly rely on a 1D respiratory surrogate (eg, 1D diaphragmatic MR navigator signals, respiratory bellows) being available during the whole PET scan. These approaches allow for real‐time respiratory motion estimation; however, they do not measure directly the respiratory‐induced motion of the heart, so that they are susceptible to changes in the breathing pattern of the patient during the acquisition. This problem is alleviated when using retrospectively gated methods that acquire 3D MRI simultaneously with the PET data. These methods use a 1D diaphragmatic MR navigator or self‐gating signals to assign the MR data to a number of respiratory windows or bins, so that each of these bins contain data acquired at a similar respiratory position throughout multiple breathing cycles. Registration of MR images obtained for each bin allows estimation of bin‐to‐bin respiratory motion that can then be used to correct the equally binned PET data. However, these methods neglect any intrabin motion and prohibit the acquisition of diagnostic MR images during the PET scan. Furthermore, to our knowledge, both precalibrated motion models and retrospectively gated approaches have used the MR‐measured motion fields only to correct the PET data, but not to improve the MR images. The MR acquisition performed before or simultaneously with PET is usually designed only to estimate motion fields, but it is not part of the diagnostic MR protocol, leading to long acquisition times, given that the required diagnostic MR images need to be acquired after the simultaneous PET‐MR acquisition.

Here, we propose a framework to correct for nonrigid respiratory motion in simultaneously acquired CMRA and cardiac PET data, allowing visualization of the coronary arteries and quantification of myocardial viability (or perfusion) in a single examination. This is achieved by combining a beat‐to‐beat image navigator (iNAV)‐based 2D translational motion correction with a bin‐to‐bin 3D nonrigid motion correction for CMRA on a 3 Tesla (T) hybrid PET‐MR system, so that MR‐derived nonrigid motion fields are used to correct for both the CMRA and the simultaneously acquired PET data. The beat‐to‐beat 2D translational motion correction allows for compensating the main components of intrabin motion, whereas 3D nonrigid deformations of the heart during the breathing cycle are captured by the bin‐to‐bin motion correction. This approach is highly efficient because nearly all acquired MR data are used for reconstruction (∼100% scan efficiency after outlier rejection), resulting in a shorter scan time than conventional gated MR acquisitions, as recently demonstrated for coronary and vessel wall balanced steady‐state free precession (bSSFP) MR imaging at 1.5T 27. Additionally, it allows for the acquisition of diagnostic MR and PET images simultaneously, significantly reducing the total exam time compared to techniques that perform diagnostic MR acquisitions after the PET scan. The framework for motion‐corrected CMRA reconstruction was tested in 10 healthy subjects and compared against a reference scan with diaphragmatic navigator gating and tracking. The MR‐based PET motion correction approach was tested in 5 oncology patients (without known or suspected CAD) with radiotracer (^18^F‐FDG) uptake in the myocardium.

## METHODS

### Image Acquisition

**Figure 1:**
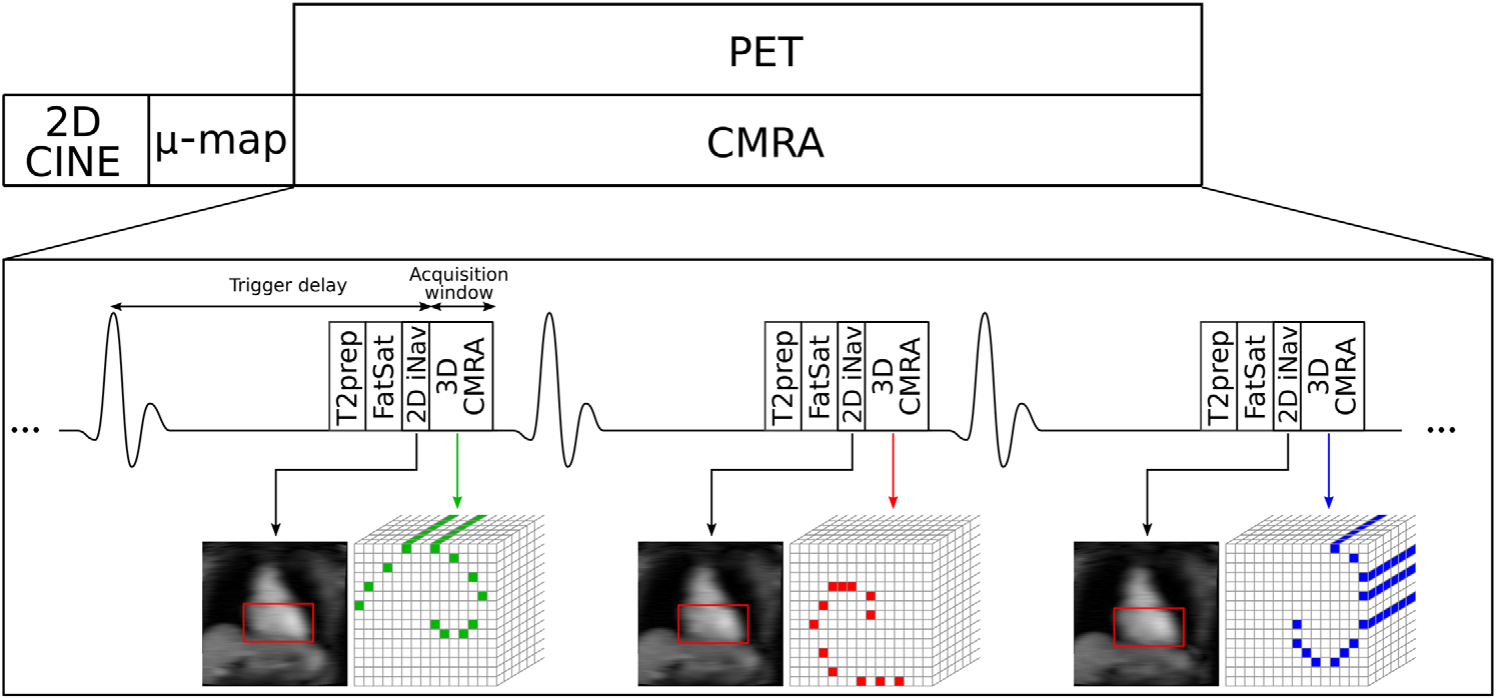
Simultaneous cardiac PET‐MR acquisition scheme. Before the CMRA‐PET acquisition, a μ‐map is acquired for attenuation correction of the PET data and a 2D CINE image is acquired to define a subject‐dependent trigger delay and acquisition window for the CMRA acquisition. 3D CMRA data are acquired using a golden‐step Cartesian spiral profile order sampling trajectory (one spiral interleaf per heartbeat), and a low‐resolution 2D image navigator (2D iNAV) is acquired at each cardiac cycle using spatially encoded low flip angle start‐up echoes at the beginning of the CMRA acquisition. T_2_ preparation (T_2_prep) and fat saturation (FatSat) pulses are applied before CMRA acquisition to improve contrast between the coronary arteries and the surrounding tissues. List‐mode PET data are acquired during the whole CMRA acquisition.

The proposed PET‐MR acquisition protocol consists of an electrocardiogram (ECG)‐triggered free‐breathing CMRA acquisition simultaneously acquired with myocardial viability list‐mode PET data on a 3T hybrid PET‐MR system. Before the simultaneous PET‐MR acquisition, a standard Dixon‐based attenuation map (µ‐map) is acquired during breath‐hold at end expiration 28 and a 2D breath‐hold CINE acquisition is performed to determine a subject‐dependent trigger delay and acquisition window for CMRA, as shown in Figure 1.

The CMRA data are acquired using a 3D T_1_‐weighted spoiled gradient echo sequence with a fully sampled golden‐step Cartesian spiral profile order sampling trajectory 29, with one spiral interleaf acquired at each cardiac cycle. A low‐resolution 2D iNAV is acquired at each cardiac cycle by adding spatially encoded low flip‐angle lines at the beginning of each interleaf of the spoiled gradient echo CMRA acquisition, in contrast to our previous approaches where the start‐up echoes of a bSSFP acquisition were spatially encoded to obtain the iNAVs 30. An adiabatic T_2_ preparation pulse is performed before data acquisition at each heartbeat to improve contrast between blood and myocardium without the use of exogenous contrast agents 31.

### Motion Estimation and Motion‐Corrected MR Reconstruction

**Figure 2:**
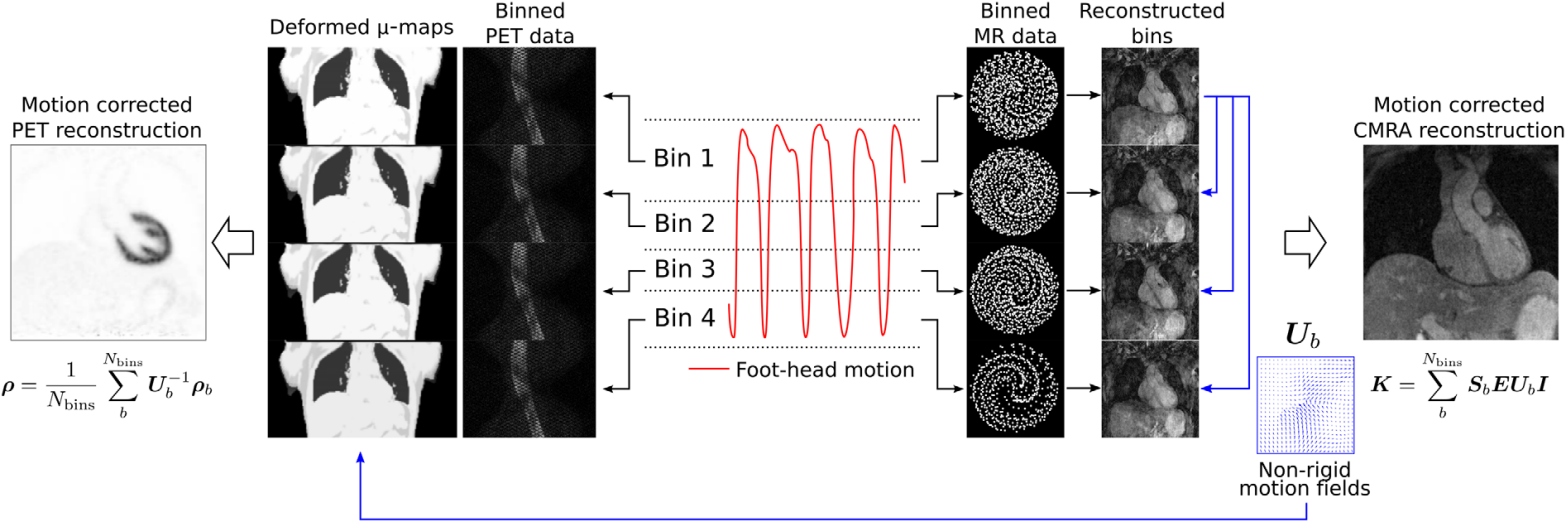
Motion‐corrected PET‐MR reconstruction scheme. The foot‐head translational motion estimated from the iNAVs, acquired at each cardiac cycle, is used to bin the acquired MR and PET data into different respiratory windows. Reconstructed MR images at each respiratory position are used to estimate 3D nonrigid motion fields that are then used to correct both PET (emission and attenuation) and CMRA data.

MR motion estimation and correction is performed in a beat‐to‐beat and bin‐to‐bin fashion for gradient echo acquisition at a 3T PET‐MR scanner, similarly to what we have previously introduced for bSSFP CMRA at 1.5T 27. First, beat‐to‐beat 2D translational motion in the foot‐head (FH) and right‐left (RL) directions is estimated from the iNAVs using normalized cross‐correlation of a template covering the apex of the heart (Fig 1), using the first acquired 2D iNAV as a reference frame. The estimated FH motion is used to assign the CMRA data to *N*_*bins*_ different respiratory bins according to their position in the breathing cycle, as shown in Figure 2. Outlier data attributed to deep breaths are removed, so that data acquired within 2 standard deviations (SDs) from the mean FH translation are used for reconstruction. MR data assigned to each respiratory bin are corrected for 2D translational motion to the center of the bin by applying a phase shift in k‐space according to the estimated FH and RL motion. The bins are then reconstructed using the iterative SENSE 32 soft‐binning approach where the acquired data are weighted according to its FH distance to the center of the bin by solving Equation [1]:

(1) $${\hat{I}}_{b}=arg\;min_{I_{b}}\left\{\|W_{b}{\left(EI_{b}-K_{b})\right\|}_{2}^{2}\right\},\;\;b=1\ldots N_{bins}$$

where ${\hat{I}}_{b}$ are the reconstructed bin images, $W_{b}$ is a diagonal matrix containing data weights for bin *b*, ***E*** is the encoding matrix including the discrete Fourier transform and coil sensitivities, and $K_{b}\;$ is the data acquired at each respiratory bin after 2D translational motion correction. The diagonal elements of $W_{b}$ are defined as a function of the respiratory position where the k‐space data was acquired, so that points acquired within the bin have a unitary weight, and points acquired outside the bin have a weight that decreases linearly to zero as the distance to the bin increases. This allows the partial inclusion of data acquired outside the bin to reduce the effect of undersampling in each respiratory bin. The soft‐binning can be formulated as shown by Equation [2]:

$$w_{k}^{b}=\;\{\begin{array}{c} 1,\;\;if\;\left(D_{FH}\left(b\right)-T_{FH}^{b}\left(k\right)+\delta \right)/\delta >1\\ 0,\;\;if\;\left(D_{FH}\left(b\right)-T_{FH}^{b}\left(k\right)+\delta \right)/\delta <0\\ \left(D_{FH}\left(b\right)-T_{FH}^{b}\left(k\right)+\delta \right)/\delta ,\;otherwise \end{array}$$

where $w_{k}^{b}\;$ are the diagonal weights for data *k* at bin *b*, $D_{FH}\left(b\right)$ is the FH distance from the bin center to the edge of the bin, $T_{FH}^{b}\left(k\right)$ is the FH distance between the center of bin *b* and the respiratory position when the k‐space point *k* was acquired, and *δ* is a parameter defining the amplitude of the soft‐gate.

After each bin has been reconstructed, bin‐to‐bin 3D nonrigid motion is estimated by image registration based on free‐form deformations with normalized mutual information as similarity metric 33, using the end‐expiration bin as a reference. This algorithm outputs direct and inverse motion fields that transform an image at the reference position to any given respiratory position and vice versa, respectively. The estimated 3D nonrigid motion fields are incorporated directly into a general matrix description (GMD) reconstruction framework 27, 29, 34. Considering ***I*** as the motion‐free image, the motion‐corrupted acquired k‐space data ***K*** is given by Equation [3]:

(3) $$K=\overset{N_{bins}}{\underset{b}{\sum }}S_{b}EU_{b}I$$

where ***S***_*b*_ is the sampling matrix that selects the k‐space points acquired at bin *b*, ***E*** is the encoding matrix, and ***U***_*b*_ is the nonrigid motion fields that transform the object in the reference position to a motion state *b*. The motion‐free image is then reconstructed by solving Equation [3] for ***I*** using a linear conjugate gradient method 35.

### Motion‐Corrected PET Reconstruction

List‐mode PET data are assigned to different respiratory bins according to their position in the breathing cycle, as previously described for the CMRA data. The same bins are used for both PET and MR data, as shown in Figure 2, using the ECG signal to synchronize the PET and MR acquisitions.

Motion‐corrected PET reconstruction is performed using a postreconstruction registration approach, also known as reconstruct transform average 36. Each respiratory bin is reconstructed independently using the ordered subsets expectation maximization (OSEM) algorithm 37, including random and scatter as well as attenuation and normalization effects. OSEM is a widely used iterative PET reconstruction algorithm that divides the acquired data into a number of subsets and calculates image updates for each subset in turn. One full iteration of the algorithm comprises one loop over all the subsets. The 3D nonrigid motion fields provided by the simultaneously acquired CMRA data are used to compute one µ‐map for each respiratory bin position by transforming the static attenuation map acquired at end expiration to each motion state. Thus, each bin is reconstructed according to Equation [4]:

(4) $$\rho_{b}^{\left(it+1\right)}=\frac{\rho_{b}^{\left(it\right)}}{P^{T}A_{b}^{T}N^{T}1_{I}\;}P^{T}A_{b}^{T}N^{T}\frac{y_{b}}{NA_{b}\rho_{b}^{\left(it\right)}+r+s}$$

where $\rho_{b}^{\left(it\right)}$ is a vector that contains the PET image for bin *b* after *it* iterations of the algorithm, ***P*** is a matrix that models the geometric aspect of the system forward projection, ***N*** and $A_{b}\;$ are diagonal matrices with entries down the diagonal equal to the reciprocal of the normalization and attenuation correction factors for bin *b*, respectively, $y_{b}$ is a vector that contains the data acquired in bin *b*, and ***r*** and ***s*** represent estimations of random and scattered coincidences, respectively. For simplicity, the subsets division was omitted in Equation [4]. It is worth noting that, in general, background (ie, random and scatter) coincidences are assumed to vary slowly compared to the activity distribution, so that the effect of motion on them is neglected.

Once all bin images have been reconstructed, they are transformed back to the reference end‐expiration bin using inverse motion fields and ultimately averaged so that one motion compensated image is obtained using all measured data as follows (Eq. [5]):

(5) $$\rho =\frac{1}{N_{bins}\;}\overset{N_{bins}}{\underset{b=1}{\sum }}U_{b}^{-1}\rho_{b}$$

As can be observed in Equation [5], the motion operators are applied to the data in PET image space at each respiratory position. Because the MR motion fields are estimated at a higher spatial resolution, they are resampled before PET reconstruction.

### Experiments

In vivo experiments were performed in healthy subjects and oncology patients without known or suspected CAD on a 3T hybrid PET‐MR scanner (Biograph mMR; Siemens Healthcare, Erlangen, Germany). Written informed consent was obtained from all subjects according to institutional guidelines, and the institutional ethics committee approved the study.

### Healthy Subject Data Acquisition

Ten healthy subjects (aged 30.0 ± 3.7 years) were scanned during free breathing using a prototype implementation of the proposed CMRA sequence. The following acquisition parameters were used: coronal slices; RL phase encoding; 1 × 1 mm^2^ in‐plane resolution; 304 × 304 in‐plane matrix size; 2‐mm slice thickness; subject‐specific number of slices covering the whole heart ranging from 40 to 48 slices; repetition time (TR)/echo time (TE) = 3.7/1.7 ms; flip angle = 15 °; and readout bandwidth (per pixel) = 685 Hz. A subject‐specific trigger delay was set targeting the mid‐diastolic rest period, and an acquisition window ranging from 89 to 119 ms (corresponding to 24–32 k‐space lines acquired per heartbeat) was used depending on the length of the diastolic period of the subject. In order to improve the contrast between the coronary arteries and the surrounding tissue, an adiabatic T2 preparation pulse 31 of 50 ms was implemented and a fat saturation prepulse was used. For the 2D iNAV acquisition, the following parameters were used: centric‐out Cartesian trajectory; coronal orientation; RL phase encoding; flip angle = 3 º; and 14 lines 30 with the same field of view (FOV) of CMRA acquisition (304 mm × 304 mm), obtaining a 1 × 21.7 mm^2^ acquired in‐plane resolution, interpolated to 1 × 1 mm^2^ reconstructed resolution, with a slice thickness ranging from 80 to 96 mm, depending on the coverage of the CMRA acquisition.

An additional Cartesian ECG‐gated reference CMRA scan with a 1D diaphragmatic respiratory gating (6‐mm gating window) and tracking (tracking factor of 0.6) with the same acquisition parameters was performed for comparison purposes 38. In order to avoid long scan times, the ECG‐gated acquisition was performed using generalized autocalibrating partially parallel acquisition parallel imaging 39 with an undersampling factor of 2 and 24 calibration lines.

### Patient Data Acquisition

Five oncology patients (aged 53.4 ± 10.4 years) received an FDG injection of 334.82 ± 24.35 MBq and were scanned 2.3 ± 0.5 hours postinjection. Before the CMRA acquisition, a µ‐map was acquired during a 19‐second breath‐hold at end expiration using the vendor's standard Dixon acquisition (imaging parameters: coronal orientation; FH phase encoding; TR/TE1/TE2 = 3.60/1.23/2.46 ms; 328 × 500 × 399 mm^3^ FOV, and 2.604 × 2.604 × 3.12 mm^3^ resolution 28). Following this, subjects were scanned during free breathing using the proposed CMRA sequence with the same acquisition parameters described for the healthy subjects. List‐mode PET data were acquired during the whole CMRA acquisition.

### MR Reconstruction

MR image reconstruction was performed inline in the scanner software for 2D translational motion correction only and offline in MATLAB (The MathWorks, Inc., Natick, MA, USA) for the 2D translational and 3D nonrigid motion correction approach. The CMRA data acquired with the proposed approach were reconstructed with 1) the proposed approach (TC+GMD), 2) 2D translational motion correction only (TC), and 3) without motion correction (no motion correction; NMC) for comparison purposes. The proposed method used between three and six respiratory bins automatically defined, such that there is a maximum bin size of 4.5 mm and each bin had approximately the same amount of data 29. The soft‐gate amplitude (*?)* was empirically determined and set to 1 mm. The TC+GMD required 2D translational motion‐corrected soft‐gated iterative sensitivity encoding reconstructions (reconstruction time of ∼250 seconds per bin), followed by 3D bin‐to‐bin nonrigid registration (reconstruction time of ∼50 seconds per bin) and, finally, the GMD reconstruction (reconstruction time of ∼2,200 seconds), for a total reconstruction time of 3,700 seconds. CMRA data acquired with 1D diaphragmatic respiratory gating and tracking (Gated) were also reconstructed inline in the scanner software for comparison purposes.

### PET Reconstruction

List‐mode PET data were binned using the MR‐derived respiratory signal, so that one 3D set of span 11 sinograms per respiratory bin was created. Nonrigid motion fields estimated from MR were used to deform the µ‐map acquired at end expiration to each respiratory bin position. PET image reconstruction was performed separately for each respiratory bin in Siemens e7 Tools, using the OSEM algorithm with three iterations and 21 subsets, including point‐spread function modeling. Images were reconstructed with a voxel size of 2.08 × 2.08 × 2.03 mm^3^ and a matrix size of 344 × 344 × 127. Once all bins were reconstructed, they were transformed to the reference respiratory position and averaged in MATLAB (The MathWorks, Inc.) to produce one motion‐corrected image (MC). The MC reconstruction required a set of OSEM reconstructions (∼240 seconds per bin), followed by 3D nonrigid deformation (∼10 seconds per bin) and finally averaging, for a total reconstruction time of 1,250 seconds. Additionally, 1) an uncorrected reconstruction including all the acquired PET data (NMC) and 2) a gated reconstruction at end expiration (Gated) were performed for comparison purposes.

### Image Analysis

In order to evaluate the quality of motion correction, each reconstructed MR image was reformatted onto a 2D plane containing both the right coronary artery (RCA) and left anterior descending (LAD) artery using dedicated software 40. Metrics of vessel length and sharpness were obtained separately for the first 4 cm and for the whole visible length of each coronary artery. Vessel sharpness values were normalized to the signal intensity of the center line of each vessel, so that 100% sharpness refers to a maximum signal intensity change at the vessel edge. For the healthy subjects, the difference between the image quality metrics (vessel length and sharpness) for each reconstructed image was evaluated with a paired *t* test with a *P* value of 0.01 considered statistically significant, including corrections for multiple comparisons.

Reconstructed PET images were visually assessed with line profiles across the left ventricle. Additionally, the mean and coefficient of variation (CV) of the standardized uptake value (SUV) in a region of interest (ROI) within the myocardium were analyzed for all reconstructed PET images. The ROI was defined as a 10‐mm‐diameter sphere inside the myocardium. Because the ROI is close to the edge of the myocardium, the mean value reflects the sharpness of images, because blurring will induce a decrease in SUV. On the other hand, the CV (SD/mean) is conventionally used to measure noise levels in PET imaging 41. When high levels of noise are present in reconstructed PET images, a postprocessing Gaussian filter is typically applied to reduce the noise level at the expense of introducing some blurring. In order to evaluate the improvement achieved by the motion correction, analysis was performed here in the original unsmoothed images and in the postprocessed images smoothed with a 4‐mm Gaussian filter.

## RESULTS

### Healthy Subjects

Scans were completed successfully in all subjects. The average acquisition time for the proposed CMRA acquisition was 12.3 ± 1.7 minutes compared to 17.0 ± 3.2 minutes for the 2 × accelerated acquisition with 1D navigator gating and tracking. The minimum, maximum, and average (and SD) efficiency of the latter was 39%, 65%, and 52.3 ± 8.9%, respectively, for a 6‐mm gating window. For the proposed approach, three to five (4.3 ± 0.9 bins, on average) respiratory bins were used with bin sizes of 2.84 ± 1.20 mm, on average.

**Figure 3:**
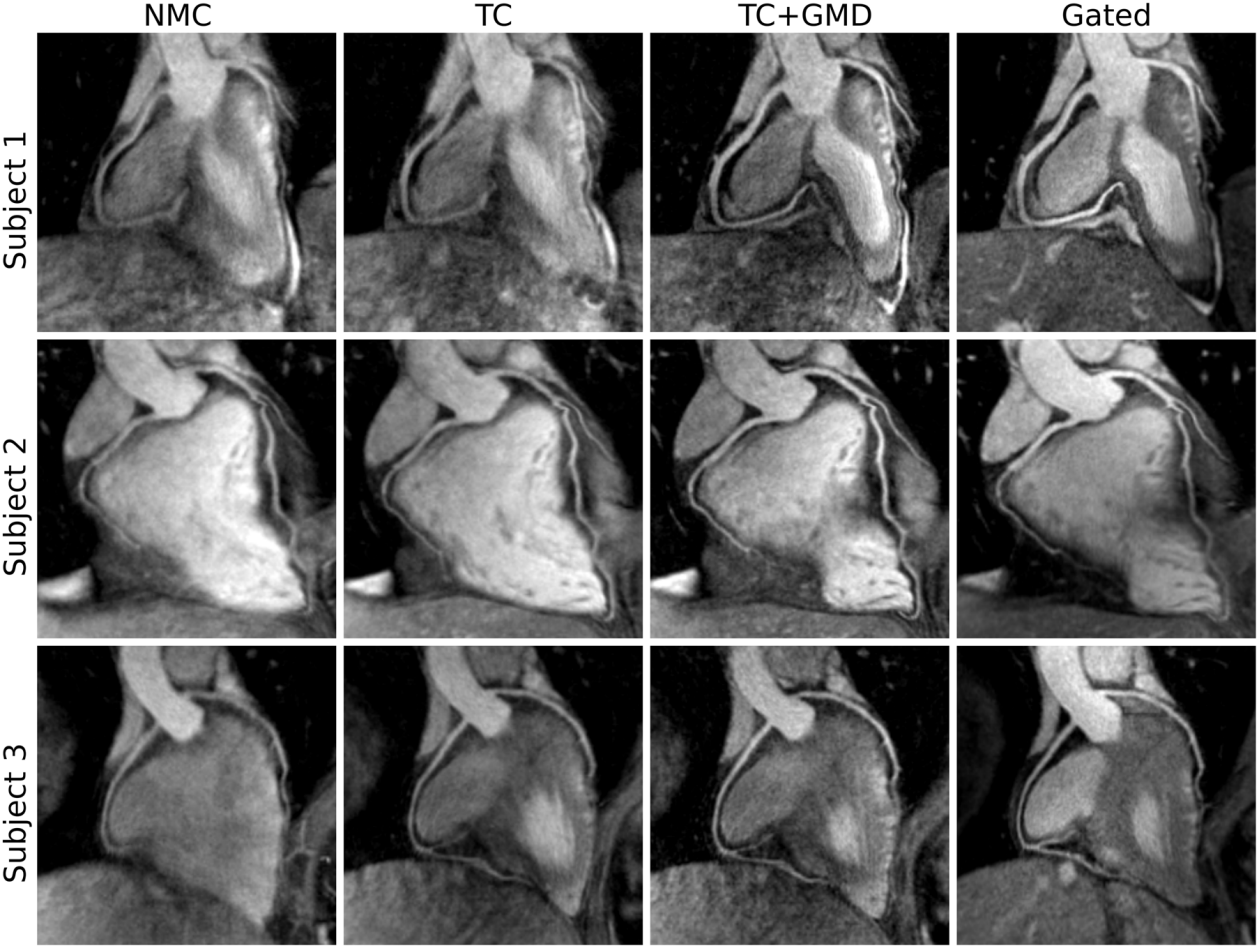
Reformatted images for 3 representative healthy subjects (rows) showing NMC, translational motion corrected (TC), translational plus nonrigid motion‐corrected (TC+GMD), and gated and tracked (Gated) images. Improvements in the visualization of the distal part of the RCA and LAD can be observed when applying TC and TC+GMD in comparison to NMC. Because the motion is tracked near the apex of the heart (Fig 1, in red over iNAV), TC produces a loss in definition of the proximal LAD for subject 1. The TC+GMD approach produces images of quality comparable to the Gated images.

Figure 3 shows the reformatted images for the NMC, translational motion‐corrected (TC), and nonrigid motion‐corrected (TC+GMD) reconstructions for 3 representative subjects. Gated images are also displayed for comparison purposes. Improvements in the visualization of the distal part of the LAD and RCA can be observed for all subjects when applying TC in comparison with NMC; further improvements can be observed with TC+GMD in the visualization of the vessels. Similar image quality can be observed for TC+GMD and Gated approaches for both LAD and RCA coronary arteries. Loss in vessel definition and sharpness can be observed in the proximal LAD for subject 1 with TC. TC correction depends on the position of the template for motion tracking (given that the motion of the heart is not purely translational), which, in this work, was located near the apex of the heart apex (Fig 1, in red over iNAV). The TC+GMD approach overcomes this limitation by correcting for the complex nonrigid motion of the heart.

**Figure 4:**
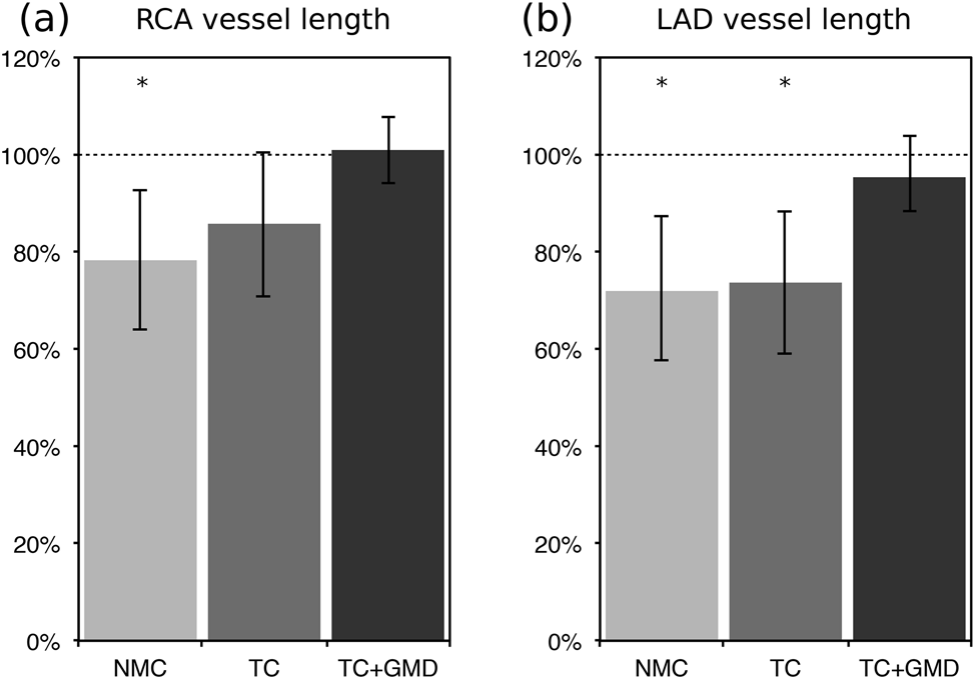
Vessel length along the RCA (**a**) and LAD (**b**) arteries for 10 healthy subjects for NMC, TC, and TC+GMD. Each measure is normalised to the length observed in the corresponding Gated image. *Statistically significant difference with *P* < 0.01 compared to the Gated images.

**Figure 5:**
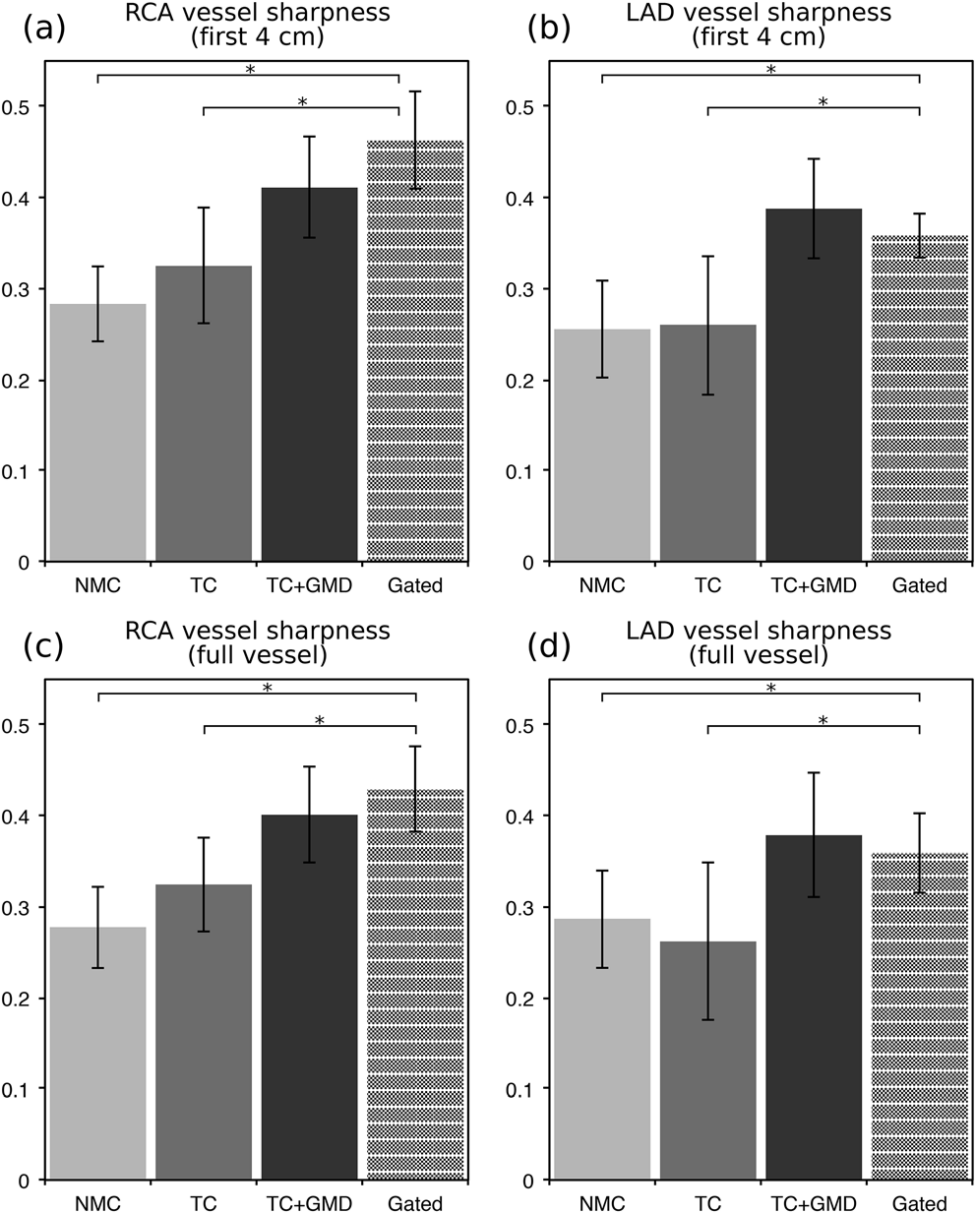
Vessel sharpness of the RCA and LAD for 10 healthy subjects for NMC, TC, TC+GMD, and Gated. (**a,c**) Vessel sharpness for the first 4 cm (**a**) and full length (**c**) of the RCA. (**b,d**) Vessel sharpness for the first 4 cm (**b**) and full length (**d**) of the LAD. *Statistically significant difference with *P* < 0.01 compared to the Gated images.

Image quality metrics for the RCA and LAD are displayed in Figures 4 and 5. It can be observed in Figure 4 that measured vessel lengths were similar for TC+GMD and Gated, reaching 101.0 ± 6.9% and 95.4 ± 8.7% of the visible length of the Gated images for the RCA and LAD, respectively. Lower values were obtained for TC and NMC, with an average of 85.7 ± 14.9% and 78.3 ± 14.2% for the RCA and 73.7 ± 14.7% and 72.0 ± 15.6% for the LAD, respectively. Significant differences were found between Gated and NMC for both coronaries and between Gated and TC for the LAD. Similar results were obtained for vessel sharpness, as can be observed in Figure 5, with significant differences between Gated and NMC and between Gated and TC for both LAD and RCA coronaries, when analyzing the proximal segment (first 4 cm) or the full length of each vessel. No statistically significant differences were observed between the Gated and the TC+GMD approach for any of the measurements.

### Patients

**Figure 6:**
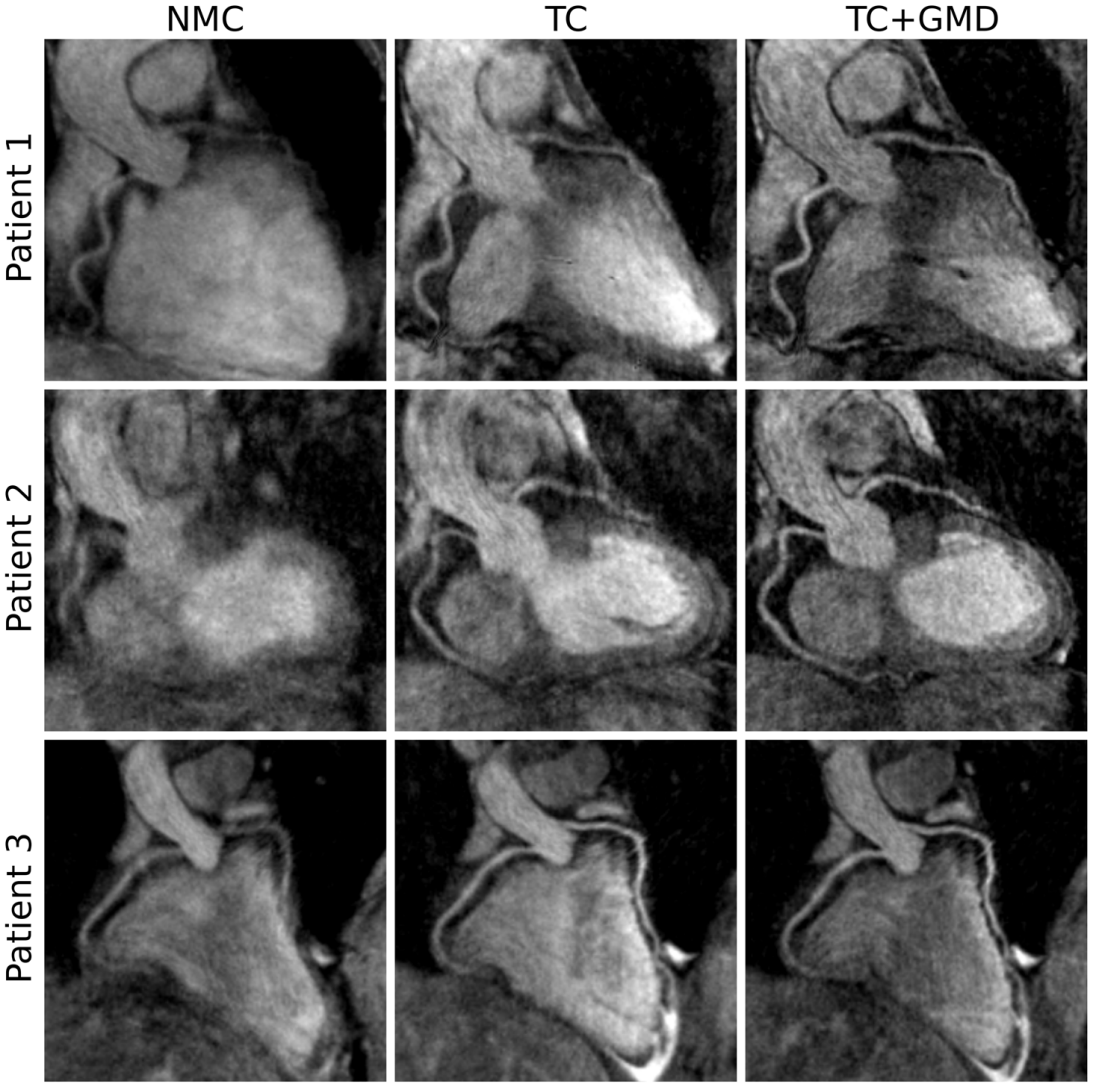
Reformatted images for 3 representative oncology patients (rows) showing NMC, TC, and TC+GMD images. Extreme motion prevents visualizing the LAD in patient 2. Improvements in the visualization of the vessels are observed when applying TC, and further improvements are observed with TC+GMD in all cases.

Scans were completed successfully in all subjects. The average acquisition time for the proposed CMRA acquisition was 10.8 ± 0.7 minutes. For the TC+GMD reconstruction, three to six respiratory bins (4.4 ± 1.5 bins, on average) were used with bin sizes of 2.77 ± 1.06 mm, on average. Figure 6 shows the reformatted images for the NMC, TC, and TC+GMD reconstructions for three representative patients. Pronounced blurring is observed in all cases when no motion correction is applied; extreme blurring prevents visualizing the proximal LAD for patient 2. TC reconstructed images allow for visualization of the proximal segments of the vessel in all the cases; however, important residual aliasing still can be observed. A consistent improvement in the visualization of the distal segment of the coronaries can be observed for all subjects when applying TC+GMD.

**Figure 7:**
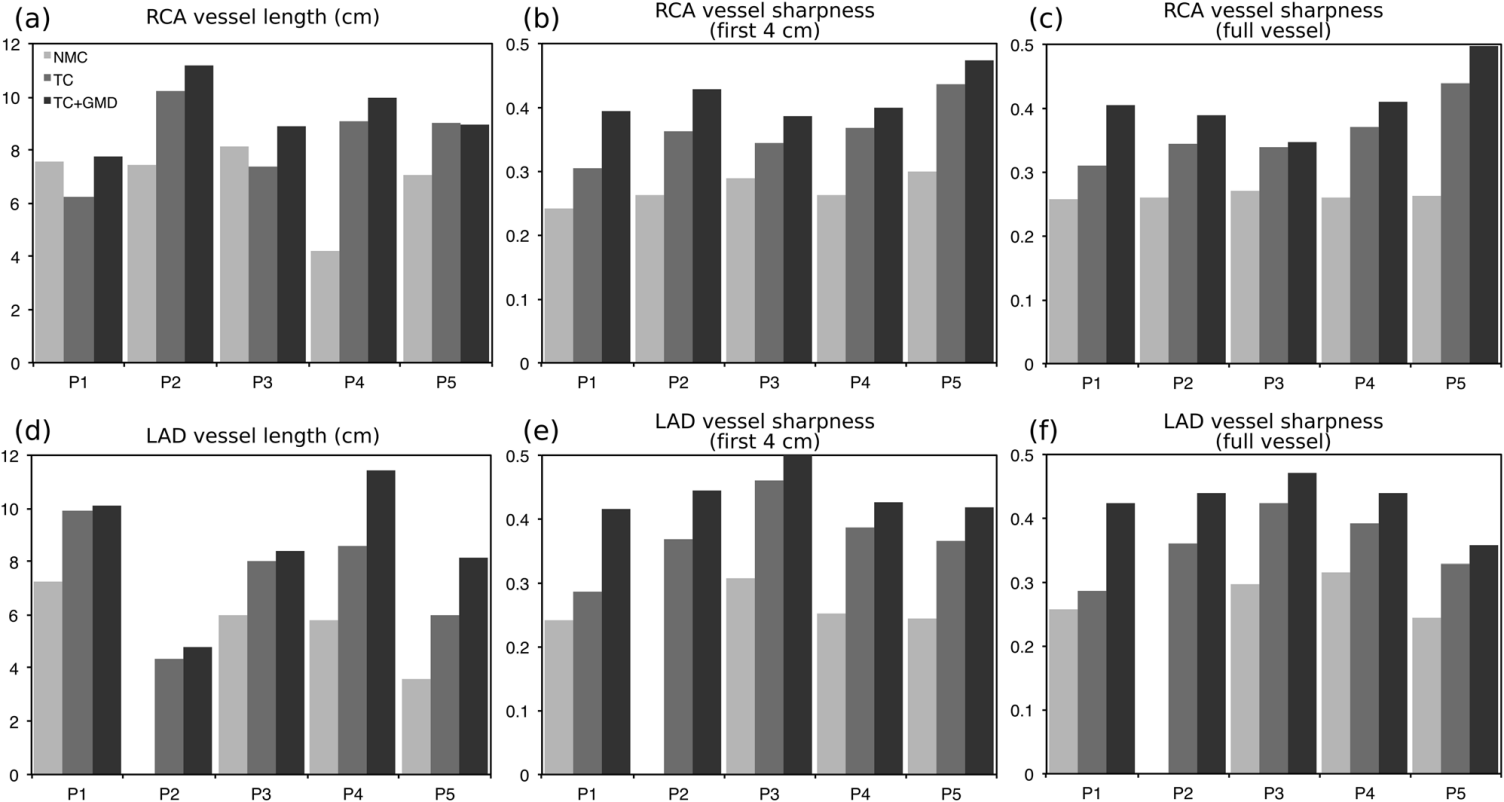
Image metrics for the RCA and LAD arteries for 5 oncology patients for NMC, TC, and TC+GMD. (**a,d**) Vessel length along the RCA (**a**) and LAD (**d**). (**b,c**) Vessel sharpness for the first 4 cm (**b**) and full length (**c**) of the RCA. (**e,f**) Vessel sharpness for the first 4 cm (**e**) and full length (**f**) of the LAD.

Image quality metrics confirm the observed improvements in sharpness for both the RCA and the LAD for all the subjects (P1–P5), as shown in Figure 7b,c and 7e,f. The visible length of the coronaries also increases when applying TC+GMD compared to TC and NMC. On average, visible length of the RCA and LAD increased by 45.53% and 75.45%, respectively, when applying TC+GMD compared to NMC images. Similarly, vessel sharpness for the full visible length of the RCA and LAD increased, respectively, by 56.44% and 51.11% on average.

**Figure 8:**
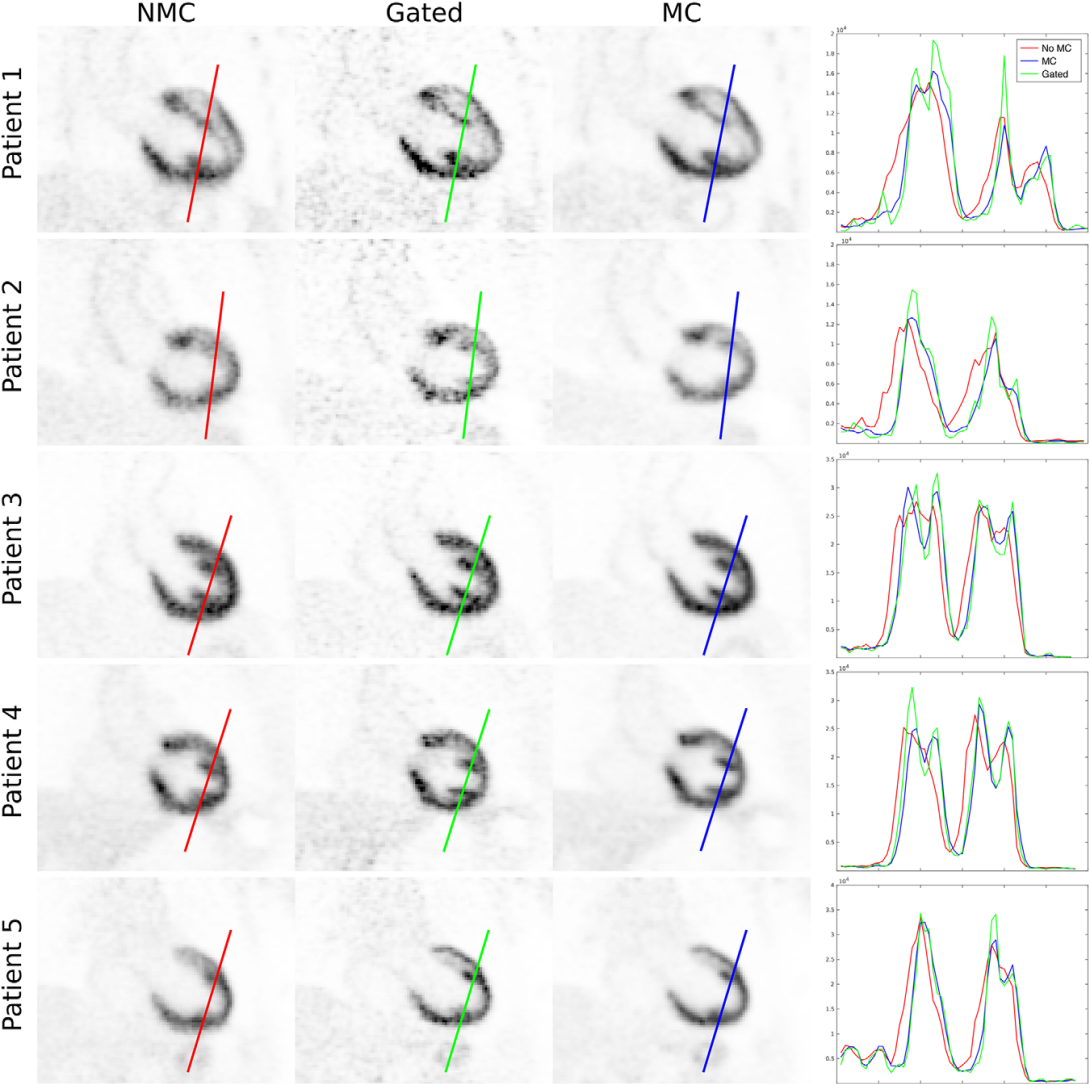
Coronal slice for 5 oncology patients (rows) showing NMC, Gated, and MC PET images, alongside with profiles across the myocardium. MC improves the sharpness of the myocardium compared to NMC and reduces noise compared to Gated.

**Figure 9:**
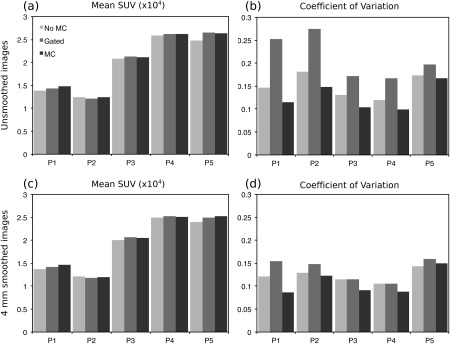
Mean and CV of the SUV for NMC, Gated, and MC PET images in a spherical ROI within the myocardium of 10‐mm diameter. Each reconstructed image was smoothed after reconstruction with a 4‐mm Gaussian filter, and both the unsmoothed and smoothed images were analyzed. For all patients, MC outperforms Gated and NMC in terms of noise (ie, less CV) and outperforms NMC in terms of mean SUV, suggesting an increased sharpness.

Reconstructed PET images with NMC, Gated (only considering the end‐expiration respiratory bin, containing 27.6 ± 8.4% of the acquired data), and MC for patients 1 to 5 are shown in Figure 8. Profiles across the myocardium are also displayed. It can be observed that MC improves sharpness of the myocardium compared to NMC while reducing noise compared to the Gated reconstruction. From the shown profiles, it can be observed that small features, such as the papillary muscles, cannot be identified in the NMC images. In the Gated images, sharpness is improved at the expense of an increased noise, and small features become apparent. On the other hand, profiles across MC images show the same sharpness as the Gated images while keeping signal‐to‐noise levels comparable to NMC. Figure 9 shows the mean SUV and the CV for each patient, for unsmoothed and smoothed images. It can be observed than when no smoothing is applied, the mean SUV in the ROI for NMC is lower than in the Gated images, suggesting that NMC is underestimating the real uptake value attributed to blurring. When MC is used, the mean increases for 3 of the patients, and it remains nearly constant for the other 2 patients. MC images also have a lower CV compared to Gated images for all the subjects. When images are smoothed with a 4‐mm Gaussian filter, it can be observed that the CV is significantly reduced for Gated images, but the minimum CV is still achieved by the MC images.

**Figure 10:**
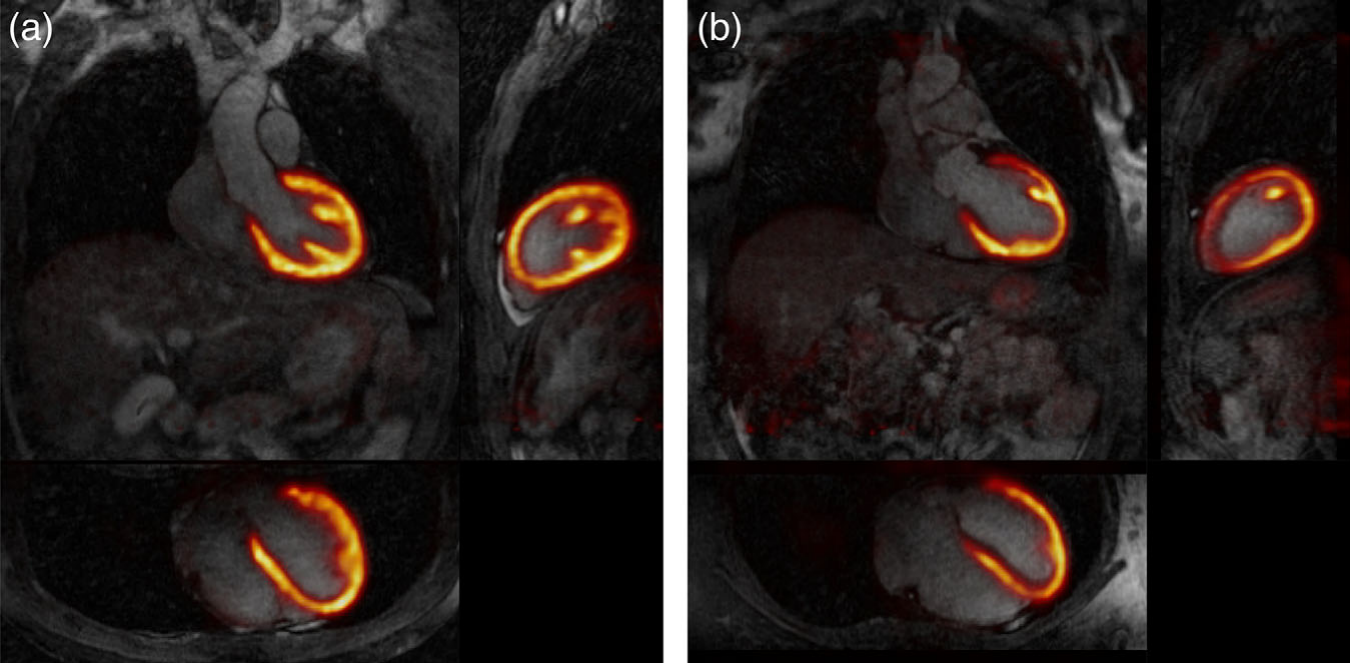
Example coronal, sagittal, and transverse views of fused motion compensated cardiac PET‐CMRA images for (**a**) patient 3 and (**b**) patient 5.

Figure 10 shows example slices for 2 representative patients of the fused motion‐compensated PET‐CMRA image. It can be observed that the motion compensation scheme produces coregistered images at the same respiratory position, and the left ventricle myocardium matches in both modalities.

## DISCUSSION

A respiratory motion‐corrected approach for simultaneous whole‐heart coronary MR angiography and cardiac PET imaging has been proposed and implemented for simultaneous coronary lumen and myocardial viability imaging. The proposed method estimates FH and RL translational motion with a high temporal resolution in a beat‐to‐beat fashion and uses the FH motion to bin both the acquired CMRA and PET data in several respiratory bins and to correct the CMRA data for intrabin motion. MR images reconstructed for each bin are used to estimate 3D nonrigid bin‐to‐bin respiratory motion, which is then used to correct both the CMRA and PET data. The motion‐corrected MR reconstruction approach has been recently proposed for coronary and vessel wall imaging at 1.5T for a bSSFP sequence with a radial‐like Cartesian trajectory 27, and it is extended here to a 3T hybrid PET‐MR system using a 3D spoiled gradient echo sequence with a golden‐step Cartesian trajectory with spiral profile order sampling without the use of exogenous contrast agents.

The MR acquisition and reconstruction approach was tested in a cohort of 10 healthy subjects and compared against a 6‐mm gated and tracked acquisition. An intermediate TC approach was implemented on the scanner, showing improvements in image quality compared to NMC reconstruction, especially in the visualization of the distal RCA and LAD. However, given that the motion of the heart during the breathing cycle is not purely translational, the TC might produce a loss of definition in the proximal segment (or distal segment, depending on the location of the template to estimate the translational motion) of the vessels for subjects with a more complex nonrigid motion of the heart. The TC+GMD approach overcomes this limitation, showing further improvements compared to TC and recovering both the proximal and distal segments of the coronary arteries. When quantifying vessel length and sharpness for both the RCA and the LAD, no statistically significant differences were observed between the Gated and the TC+GMD approach. The proposed approach had a shorter and predictable scan time of ∼12 minutes (fully sampled acquisition) compared to ∼17 minutes for the 2 × undersampled gated and tracked, while simultaneously providing nonrigid motion fields for correcting PET data. It is worth noting that the fully sampled CASPR trajectory produces an elliptical sampling mask in the phase encoding plane 29, 42, further reducing scan time compared to traditional fully sampled Cartesian acquisitions.

The full PET‐MR acquisition and reconstruction approach was then tested in a cohort of 5 oncology patients that exhibit radiotracer (^18^F‐FDG) uptake in the myocardium. For the CMRA data, extreme blurring was observed when no motion correction is applied, in some cases completely obscuring the visualization of the vessels. TC images allowed for visualization of the arteries in all cases, and a further improvement in visible length and sharpness of the coronary arteries was observed when using TC+GMD approach. Nonrigid motion fields obtained from CMRA data were also used to correct PET data for respiratory motion. Increased sharpness of the myocardium and improved visualization of small features, such as the papillary muscles, can be observed for all the subjects compared to NMC PET images. In addition, reduced noise levels compared to gated PET reconstruction were observed, because a greater number of counts are used to produce the motion‐compensated images. For reconstructed images without smoothing, an increased mean SUV in an ROI within the myocardium was observed for 3 of the subjects when applying motion correction compared to NMC images, attributable to a significant reduction in blurring. For the other 2 subjects, the value remained approximately constant, because the ROI was located in a region with less motion. When images are smoothed, it can be observed that noise levels are significantly reduced for all the images; however, minimum noise is achieved by the motion‐corrected images for all patients. Motion‐corrected images also present a larger mean SUV in the ROI compared to NMC, suggesting an increased sharpness in the image.

In this work, each PET respiratory bin was reconstructed independently, and motion fields were then used to warp all the images to a common reference position. This motion compensation approach has been shown to produce an increased bias in the standardized uptake values attributed to the reduced number of counts in each respiratory bin in simulation studies 12. Including the motion directly in the system model of the PET reconstruction algorithm can alleviate this problem, so that a motion‐compensated image reconstruction can be performed instead; however, this approach requires a longer reconstruction time. In this work, only respiratory motion correction is being performed for PET. Future work includes extending this approach for acquiring multiple cardiac phases so that both respiratory and cardiac motion correction can be performed, further reducing blurring of the myocardium in the PET images.

Translational correction for the CMRA acquisition was implemented in the scanner software so that a first correction can be immediately visualized after scanning. However, both the TC+GMD and the motion‐compensated PET reconstruction are performed offline. Future work includes implementing the whole motion compensation framework on the scanner, including PET and MR synchronization, as well as nonrigid motion estimation from MR and motion‐corrected reconstruction for both PET and CMRA. Although the respiratory motion correction scheme was compared against a gated and tracked acquisition for healthy subjects, such validation was not performed in patients, where respiratory motion can be more variable. Future studies will validate the proposed PET‐MR method in patients with heart disease, considering both myocardial viability (^18^F‐FDG) and perfusion (^13^N‐ammonia) PET acquisitions.

## CONCLUSION

A framework for simultaneous coronary MR angiography and cardiac PET has been proposed for a hybrid 3T PET‐MR system. This approach allows the correction of both the MR and PET data for respiratory motion, producing diagnostic images that allow simultaneous visualisation of the coronary arteries, myocardial viability, and, potentially, myocardial perfusion. The motion‐corrected MR reconstruction framework was validated in healthy subjects. No statistically significant differences were found when comparing the proposed approach with a standard 1D diaphragmatic undersampled gated and tracked acquisition. Our approach, however, has a predictable scan time, was 30% faster on average, and provides motion fields for PET motion correction. The PET‐MR framework was tested in oncology patients, and results show improvements in sharpness and reductions in image noise compared to standard uncorrected and gated reconstructions, respectively. Future work includes implementing the complete reconstruction framework in the scanner software and validating our approach in cardiac patients.
